# Gemtuzumab ozogamicin for relapsed or primary refractory acute myeloid leukemia in children—the Polish Pediatric Leukemia and Lymphoma Study Group experience

**DOI:** 10.3389/fimmu.2023.1268993

**Published:** 2023-12-22

**Authors:** Katarzyna Pawinska-Wasikowska, Malgorzata Czogala, Szymon Skoczen, Marta Surman, Monika Rygielska, Teofila Ksiazek, Agnieszka Pac, Aleksandra Wieczorek, Jolanta Skalska-Sadowska, Magdalena Samborska, Jacek Wachowiak, Radoslaw Chaber, Renata Tomaszewska, Tomasz Szczepanski, Karolina Zielezinska, Tomasz Urasinski, Malgorzata Moj-Hackemer, Krzysztof Kalwak, Marta Kozlowska, Ninela Irga-Jaworska, Walentyna Balwierz, Karolina Bukowska-Strakova

**Affiliations:** ^1^ Department of Pediatric Oncology and Hematology, Institute of Pediatrics, Jagiellonian University Medical College, Krakow, Poland; ^2^ Department of Pediatric Oncology and Hematology, University Children Hospital of Krakow, Krakow, Poland; ^3^ Laboratory of Clinical Immunology, University Children’s Hospital of Krakow, Krakow, Poland; ^4^ Department of Pediatric Oncology and Hematology, Hematology Laboratory, University Children’s Hospital, Krakow, Poland; ^5^ Department of Medical Genetics, Institute of Pediatrics, Jagiellonian University Medical College, Krakow, Poland; ^6^ Department of Epidemiology and Preventive Medicine, Faculty of Medicine, Jagiellonian University Medical College, Kraków, Poland; ^7^ Department of Pediatric Oncology, Hematology and Transplantology, Poznan University of Medical Sciences, Poznan, Poland; ^8^ Department of Pediatric Oncohematology, Clinical Province Hospital of Rzeszow, Rzeszow, Poland; ^9^ Department of Pediatrics, Institute of Medical Sciences, Medical College, University of Rzeszow, Rzeszow, Poland; ^10^ Department of Pediatric Hematology and Oncology, Zabrze, Medical University of Silesia, Katowice, Poland; ^11^ Department of Pediatrics, Hemato-Oncology and Gastroenterology, Pomeranian Medical University in Szczecin, Szczecin, Poland; ^12^ Clinical Department of Pediatric Bone Marrow Transplantation, Oncology and Hematology, Wroclaw Medical University, Wroclaw, Poland; ^13^ Department of Pediatrics, Hematology and Oncology, Medical University of Gdansk, Gdansk, Poland; ^14^ Department of Clinical Immunology, Institute of Pediatrics, Jagiellonian University Medical College, Krakow, Poland

**Keywords:** acute myeloid leukemia, gemtuzumab ozogamicin, relapse, refractory, children, immunotherapy

## Abstract

**Background:**

Gemtuzumab ozogamicin (GO), one of the first targeted drugs used in oncology, consists of an anti-cluster of differentiation 33 (CD33) monoclonal antibody bound to a derivative of cytotoxic calicheamicin. After the drug withdrawn in 2010 due to a significantly higher rate of early deaths, GO regained approval in 2017 for the treatment of newly diagnosed, refractory, or relapsed acute myeloid leukemia (AML) in adults and children over 15 years of age. The objective of the study was a retrospective analysis of clinical characteristics, treatment outcomes, and GO toxicity profile in children with primary refractory or relapsed (R/R) AML treated in Poland from 2008 to 2022.

**Methods:**

Data were collected through the Polish Registry of Acute Myeloid Leukemia. From January 2008 to December 2022, 35 children with R/R AML were treated with GO in seven centers of the Polish Pediatric Leukemia and Lymphoma Study Group.

**Results:**

Most of the children (30 of 35) received only one GO cycle in combination with various chemotherapy cycles (IDA-FLA, DOXO-FLA, FLA, FLAG, and others). Eighteen children (51%) achieved complete remission (CR), 14 did not respond to treatment, and three progressed. GO therapy was followed by allogeneic hematopoietic stem cell transplantation (allo-HSCT) in 18 children in CR. The 5-year overall survival (OS) after GO therapy was 37.1% ± 8.7% for the total cohort. There was a trend toward a superior outcome in patients with strong expression of CD33 expression (over 50% positive cells) compared with that in patients with lower expression of CD33 (OS, 41.2% ± 11.9% versus 27.8% ± 13.2%; p = 0.5; 5-year event-free survival, 35.4% ± 11.6% versus 25.7% ± 12.3%; p = 0.5, respectively). Children under 15 years have better outcome (OS, 34.9% ± 10.4% versus 30% ± 14.5%, p = 0.3). The most common adverse events were bone marrow aplasia, fever of unknown origin, infections, and elevated liver enzyme elevation. Sinusoidal obstruction syndrome occurred in two children.

**Conclusions:**

The use of GO in severely pretreated children, including those under 15 years of age, with previous failure of AML treatment is a feasible and effective bridging therapy to allo-HSCT with an acceptable toxicity profile.

## Introduction

Acute myeloid leukemia (AML) is a rare disease in children, caused by uncontrolled clonal proliferation of myeloid cells. Although the prognosis in childhood AML has improved significantly in recent decades, still up to 5%–10% of children do not achieve complete remission (CR) due to resistance of leukemic cells (primary refractory disease), and 30%–50% of children in CR eventually relapse (1–7). The outcomes of refractory or relapsed (R/R) AML remain poor and depend mainly on the time of relapse, genetic profile of leukemic blasts, and early response to therapy. Therapeutic options for this group of patients are still limited. Targeted or immune-based therapies appear to be a promising, although challenging, option to further improve treatment outcomes (8–10).

Gemtuzumab ozogamicin (GO) is the first antibody drug conjugate (ADC) approved for the treatment of AML. GO consists of two compounds: an anti-CD33 monoclonal antibody (a humanized immunoglobulin class G subtype 4 antibody that specifically recognizes human CD33) and a derivative of cytotoxic calicheamicin (a semisynthetic cytotoxic antibiotic). The antigen CD33 is found on the surface of myeloid leukemic cells or immature cells of the myelomonocytic lineage, excluding hematopoietic stem cells. After GO binding to CD33-expressing cancer cells, the ADC–CD33 complex is internalized, followed by intracellular release of N-acetyl gamma calicheamicin dimethyl hydrazide. Once N-acetyl gamma calicheamicin dimethylhydrazide is activated, it causes double-stranded DNA breaks, leading to cell cycle arrest and apoptosis (11).

GO has been known since the late 1990s, and its history on the market is full of ups and downs. The first studies with GO as a single agent or in combination with chemotherapies showed its efficacy and safety in pediatric and adult patients with R/R AML, with overall response of 20%–30% (12–17). Therefore, GO was approved by the Food and Drug Administration in 2000 and then suddenly withdrawn in 2010 due to a significantly higher reported rate of early deaths and unacceptable toxicities, such as infections, major bleeding, and acute respiratory distress syndrome (18, 19). Despite the withdrawal of GO from the market, studies on its efficacy and safety continued, helping to regain its approval in 2017 for the treatment of R/R and newly diagnosed AML in adults and children (20–30). Many further studies in adults and children confirmed the efficacy and safety of GO in a single dose (3 mg/m^2^) compared with that in higher doses previously used (4–9 mg/m^2^) (20–24). The introduction of fractionated lower doses of GO allowed safe use of higher cumulative doses, leading to significant improvements in treatment outcomes and a reduction in treatment-related mortality rates, as reported in the ALFA-0701 study and its update (31, 32). GO is currently registered in the United States for use in children, whereas, in Europe, it is officially approved for the treatment of *de novo* or relapsed AML in patients over 15 years of age. Therefore, the pediatric population in Europe still needs to receive GO off-label, which, in turn, reduces the availability of the drug to them.

The aim of the presented study was a retrospective analysis of clinical characteristics, treatment outcomes, and GO toxicity profile in children with primary R/R AML treated in Poland from 2008 to 2022.

## Materials and methods

From January 2008 to December 2022, 35 children with relapsed or primary refractory AML were treated with GO in seven centers of the Polish Pediatric Leukemia and Lymphoma Study Group (PPLLSG). Data were collected through the Polish Registry of Acute Myeloid Leukemia. There were 545 children aged 0–18 years with newly diagnosed AML registered from January 2008 to December 2022 in the PPLLSG database excluding children with acute promyelocytic leukemia, myelodysplastic syndrome (MDS)-related AML, AML after cytotoxic therapy, or mixed phenotype leukemia. There were 145 (26%) relapses among 545 registered children up to the end of 2022. Finally, among 35 children, eight were initially treated according to the AML-BFM 2004 Interim, 18 according to the AML-BFM 2012 Registry, and eight according to the AML-BFM 2019 protocol, whereas one child with Down syndrome was treated according to the ML-DS 2019 protocol.

We use standard definitions for relapse, primary refractory disease, and CR. Relapse was defined as a recurrence of more than 5% unequivocal leukemic blasts in a representative bone marrow identified by microscopic and/or flow cytometry methods and/or evidence of leukemic infiltration of any site. Relapse occurring within 18 months after initial diagnosis was classified as an early relapse, whereas relapse after 18 months after diagnosis was defined as late. Primary refractory disease was defined as more than 10% morphological blasts after the first induction block (assessed between days +28 and +42) and/or >5% morphological blast after second induction or aplasia <6 weeks after the beginning of the second induction. CR was defined as less than 5% of blasts in bone marrow aspirate smears, an absolute neutrophil count of >1 × 10^9^/L, platelets of 100 × 10^9^/L in peripheral blood, and no evidence of extramedullary involvement.

In the study, the results of GO therapy were evaluated in relation to disease status, genotype, expression of CD33 on leukemic blasts, and age.

The evaluation of CD33 antigen expression was performed by multiparameter flow cytometry in one center, Laboratory of Clinical Immunology, University Children’s Hospital of Krakow. Strong expression of CD33 was defined when more than 50% of leukemic cells were positive for CD33, whereas weak expression was defined when 10%–50% of leukemic cells were positive. If less than 10% of leukemic cells were positive for CD33, then the blasts were considered CD33 negative.

Toxicities, mainly in terms of mucosal toxicity, bone marrow aplasia, liver toxicity with special respect to the development of venocclusive disease, also called sinusoidal obstruction syndrome (SOS), and other adverse reactions were analyzed in relation to each GO cycle and classified using version 5.0 of the Common Terminology Criteria for Adverse Events. All patients received standard-of-care posaconazole or voriconazole for the prevention of fungal infection and trimethoprim/sulphamethoxazole as a prophylaxis of *Pneumocystis jirovecii* infection.

The study protocol has been carried out in accordance with the Ethics Code of the World Medical Association (Declaration of Helsinki) for experiments involving humans and was approved by the Ethics Committee of Jagiellonian University (protocol code: 1072.6120.227.2022, date of approval: 12 October 2022).

### Statistical analysis

The Kaplan–Meier method was used to estimate survival probabilities. The main endpoints were event-free survival (EFS) and relapse-free survival (RFS), cumulative incidence of relapse (CIR), and overall survival (OS). The EFS was defined as the time from the date of the first dose of GO administration to the first failure event (disease progression, relapse, and death). Patients who have not had an event will be censored at their last follow-up date. The RFS was calculated from the date of CR after GO to the date of relapse. CIR was other method to estimate relapse rate. The OS was defined as the time from the date of first administration of GO to death from any cause. Patients who had not died were censored at their last follow-up date. Survival probabilities were presented along with standard errors. The subgroups were compared with a log-rank test. The significance level of 0.05 was used in all statistical tests. Statistical analyses were performed with Statistical Package for Social Science version 28.0.

## Results

### Patient characteristics

Among the 35 children evaluated in the presented study, there were 18 girls and 17 boys. The median age was 6.4 (range: 0.4–18). There were 25 children under 15 years of age. The most common subtype of AML according to the classification of French–American–British was M5 (n = 8; 22%) and M2 (n = 7; 20%). There was a patient with myeloid sarcoma and a patient with myeloid leukemia with Down syndrome (M7). In first-line treatment (AML-BFM 2004 Interim, AML-BFM 2012 Registry, and AML-BFM 2019 protocols, ML-DS 2019 protocol for children with Down syndrome), most of the patients (n = 22; 63%) were classified into a high-risk group (HR) based on poor-risk genetics or inadequate response to therapy. Eight children received allogeneic hematopoietic stem cell transplantation (allo-HSCT) in the first CR.

In AML-BFM 2004 Interim protocol, the indication for allo-HSCT from sibling donors in CR was restricted to HR patients, recommended only for HR patients with bone marrow blasts >5% after the second induction. Two children from HR treated according to AML-BFM 2004 Interim had <5% blasts at this time point. Among the remaining 20 children (out of 22), 12 were refractory to treatment and did not complete all planned therapy, including HSCT. Therefore, finally, only eight children out of the 22 HR patients received HSCT in the first CR.

The analyzed patients included 12 (34%) children with primary refractory AML and 23 (66%) with relapsed AML (16 children with the first early relapse, five with the first late, and two with the second relapse). GO was administered at different time points of salvage therapies. Most of the children (73.5%) had more than 5% leukemia blasts in the bone marrow, whereas nine patients had less than 5% before starting GO therapy. The median number of leukemic blasts was 22% (range: 0.1–99).

Of the 35 patients, 51.4% had AML with an intermediate genetic profile (genetic abnormalities classified as neither poor nor good risk). Four children had CBF (*core binding factor*) AML characterized by the presence of either t(8;21)(q22;q22) or inv(16)(p13;q22)/t(16;16), whereas 13 patients had a poor-risk genetic profile. The complex karyotype and FLT3::ITD with WT1 mutation were the most common (n = 8, 23%) among patients with poor-risk genetics. Three out of the four children with CBF-AML were diagnosed with late bone marrow relapse, whereas seven out of the 13 patients with poor risk genetics had a primary refractory disease. The characteristics of the patients are shown in **Table 1**.

**Table 1:** Patient’s characteristic at initial diagnosis.

Feature	Number	%
**Total number of patients**	35	100
**Median (range)**	Age, years	
Sex		
** Female** ** Male**	18 17	51.4 48.6
FAB classification		
** M0** ** M1** ** M2** ** M4** ** M5** ** M7** ** ML-DS** ** Myeloid sarcoma**	3 5 7 6 8 4 1 1	8.5 13.5 20 17 22.5 13.5 2.5 2.5
Risk group in first-line treatment		
** SR** ** IR** ** HR**	3 10 22	8.5 28.5 63
Disease status prior to GO		
**Refractory *de novo* AML** **1st early relapse** **1st late relapse** **2nd relapse**	12 16 5 2	34 46 14 6
Site of relapse (number of patients = 23)		
** BM** ** BM + CNS** ** BM + skin + n.VII palsy** ** BM + skin** ** BM + forearm tumor**	19 1 1 1 1	82 4.5 4.5 4.5 4.5
Percentage of blasts in bone marrow before GO		
< 5% blasts 5%–25% >25% blasts ND	9 9 16 1	26.5 26.5 47
Genetic abnormalities		
**Good risk genetics**	4	11.4
**inv(16)(p13;q22)** **t(8;21)(q22;q22)**	2 2	5.7 5.7
**Poor risk genetics**	13	37.1
**FLT3::ITD and WT1** **KMT2A::MLLT10** **KMT2::MLLT3** **Monosomy -7** **BCR::ABL1** **Complex karyotype**	5 1 2 1 1 3	14.2 2.8 5.7 2.8 2.8 8.5
No impact or unknown risk genetics		
**Genetic abnormality other than HR or SR feature**	13	37
**Normal karyotype**	5	14
Previous treatment		
**AML-BFM 2004 Interim** **AML-BFM 2012 Registry** **AML-BFM 2019** **ML-DS-2019**	8 18 8 1	23 51 23 3
HSCT prior to GO therapy (first-line therapy)		
** Yes** ** No**	8 27	33 67

ND, not done; SR, standard risk; IR, intermediate risk; HR, high risk.

### Treatment

Only one patient was treated with GO monotherapy due to early refractory relapse and poor general condition. Other patients received GO as part of the chemotherapy cycle. Most of the children were treated according to the Relapsed AML 2001/01 protocol. In total, seven patients were treated with the Ida-FLA-GO cycle and received only one GO course. Children who received two doses of GO were given Ida-FLA-GO, followed by FLA-GO (four patients) or two cycles of Ida-FLA-GO (1 child). Nineteen children (54%) received allo-HSCT after GO therapy: 18 in CR and one without CR. Details of the therapy cycles given along with GO are presented in **Table 2**. GO was administered to children primarily at a dose of 4.5 mg/m^2^ (25 children; 71%) according to the Relapsed AML 2001/01 protocol. Six children received GO at a dose greater than 4.5 mg/m^2^ (median: 5.6; range: 5–7), and four children at a dose less than 4.5 mg/m^2^ (median: 3.25; range: 3–4) (**Table 3**).

**Table 2:** Treatment administered with gemtuzumab ozogamicine (GO).

Disease prior to GO	Refractory *de novo* AML n = 12	1st early relapse n = 16	1st late relapse n = 5	2nd relapse n = 2	Total n = 35
Chemotherapy before and with GO					
**Ida-FLA-GO; FLA-GO**	3	1	0	0	4
**Ida-FLA; FLA-GO**	1	2	3	0	6
**Ida-FLA; FLAG-GO**	1	0	0	0	1
**2xIda-FLA-GO**	0	0	0	1	1
**Ida-FLAG; Ida-FLAG-GO**	1	0	0	0	1
**Ida-FLA-GO**	0	6	1	0	7
**Ida-FLAG-GO**	0	0	0	1	1
**FLA-GO**	2	1	0	0	3
**TVTC, FLA-GO**	1	0	0	0	1
**Doxo-FLAG-GO**	2	1	1	0	4
**MIDAM-GO**	1	0	0	0	1
**Ida-FLA, 2CDA-Topotecan-GO**	0	1	0	0	1
**Cladribine-Topotecan-GO**	0	1	0	0	1
**Clofarabine-VP-16-Cyclofosfamide-GO**	0	1	0	0	1
**Azacytidine-GO**	0	1	0	0	1
**Monotherapy with GO**	0	1	0	0	1

Ida, idarubicin; FLA, fludarabine; GO, gemtuzumab ozogamicine; FLAG, fludarabine + GCSF; TVTC, topotecan, vinorelbine, thiotepa, and clofarabine; Doxo, doxorubicine; MIDAM, mitoxantrone, cytarabine.

**Table 3:** Details and outcomes of therapies with gematuzumab ozogamicine (GO).

Disease prior to GO	Refractory *de novo* AML	1st early relapse	1st late relapse	2nd relapse	Total
**Number of patients (%)**	12 (34)	16 (46)	5 (15)	2 (5)	35 (100)
Age in years, median (range)					
- **At initial diagnosis** - **At GO therapy**	12.15 (0.4–17.8) 12.85 (0.4–18.64)	3.25 (0.4–17.9) 3.62 (1.3–18.83)	7.0 (1.5–16.7) 7.92 (4.26–17.8)	8.95 (2.5–15.4) 9.65 (3.5–15.79)	6.6 (0.4–17.9) 7.05 (1.06–18.83)
Number of GO cycles					
- **One** - **Two**	9 3	15 1	5 0	1 1	30 5
GO dose					
**3 mg/m^2^** **4.5 mg/m^2^** **>4.5 mg/m^2^**	1 8 3	1 13 2	1 4 0	1 0 1	4 25 6
HSCT after GO					
**Yes** **No**	8 4	9 7	2 3	0 2	19 16
Bone marrow after GO					
**CR or CR** **without complete recovery** **No response** **Progression**	7 4 1	8 7 1	3 1 0	0 2 1	18 (51%) 14 (40%) 3 (9%)
Relapse after GO and HSCT					
**Yes**	4	5	0	0	9
Patients’ status at the end of follow-up					
**Alive** **Dead**	3 (25%) 9 (75%)	7 (44%) 9 (56%)	4 (80%) 1 (20%)	0 2 (100%)	14 (40%) 21 (60%)
Death					
**Progression**	1	1		1	3
**Progression after further relapse**	2	4	1		7
**COVID-19**	1				1
**CMV after HSCT**		1			1
**VOD/SOS**		1			1
**MOF after HSCT**	4	1			5
**MOF**	1	1		1	3

CR, complete remission; VOD, veno-oclussive disease; SOS, sinusoidal obstruction syndrome; AST, aspartate aminotransferase; ALT, alanine aminotransferase; MOF, multiorgan failure; HSCT, hematopoietic stem cell transplantation; CMV, cytomegalovirus.

### Toxicities

Among study patients, the most common adverse events were bone marrow aplasia, fever of unknown origin, infections, and elevated liver enzyme elevation. The incidence of toxicities was similar between children over and under 15 years of age (**Table 4**). All patients presented pancytopenia due to bone marrow aplasia after multi-agent chemotherapy combined with GO. Infections, mainly bacterial, were observed in 16 patients, whereas four children developed a proven invasive fungal infection (one aspergillosis and three candidiasis). Six children developed grade 4 mucositis. Ten children had a fever of unknown origin. Most of the patients (71%, 25 children) had demonstrated hepatotoxicity with elevated aminotransferase activity (grades 3 and 4). In one patient, an infusion-related reaction to GO was observed that included fever, hypotension, bronchospasm, and dyspnea. This patient had previously received GO with the Ida-FLA cycle at a dose of 4.5 mg/m^2^.

**Table 4:** Toxicities observed during therapy with gemtuzumab ozogamicine (GO).

Toxicties	Number (% of total)	<15 y n = 25 (%)	≥15 y n = 10 (%)
**Invasive fungosis**	4 (11)	1 (4)	3 (30)
**Fever of unknown origin**	10 (29)	5 (20)	5 (50)
**Infections**	16 (46)	10 (40)	6 (60)
**Mucositis, grade 4**	6 (17)	4 (16)	2 (20)
**Pancreatitis**	1 (3)	1 (4)	0
**VOD/SOS**	2 (6)	2 (8)	0
**Infusion-related/allergic reaction**	1 (3)	1 (4)	0
**Thrombosis**	1 (3)	1 (4)	0
**Neutropenia (grades 3 and 4)**	34 (97)	24 (96)	10 (100)
**Anemia (grades 3 and 4)**	32 (91)	23 (92)	9 (90)
**Thrombocytopenia (grades 3 and 4)**	33 (94)	24 (96)	9 (90)
**AST/ALT elevation (grades 3 and 4)**	25 (71)	19 (76)	6 (60)

VOD, veno-occlusive disease; SOS, sinusoidal obstruction syndrome; AST, aspartate aminotransferase; ALT, alanine aminotransferase; MOF, multiorgan failure.

SOS occurred in two children, both under 15 years. The first child revealed severe SOS (hepatomegaly, ascites, and weight gain >10%) with a fatal course. This child received GO at a dose of 4.5 mg/m^2^ along with cladribine and topotecan in the second bone marrow relapse therapy. In the treatment of the first bone marrow relapse, the patient had been transplanted. The second patient with SOS and myeloid sarcoma presented signs and symptoms of SOS on the third day after administration of GO at a dose of 3 mg/m^2^. The child presented moderate-grade SOS and responded to defibrotide therapy. The patient was treated with Ida-FLA as the first induction cycle, then received FLA and GO with good response, and proceeded to allo-HSCT. None of the 19 transplanted patients developed SOS as a sequel to allo-HSCT performed after GO therapy.

### Outcomes

The median survival time for alive patients at the last contact was 6.8 months (range: 1–148 months). More than half of the children (18) achieved CR or CR without complete hematologic recovery. Thirteen patients did not respond to GO in combination with other agents, including a patient who received GO alone. Progression was found in three children. Of the 18 patients in CR, nine relapsed.

The probability of 5-year OS after GO therapy was 37.1% ± 8.7% for the total cohort (**Supplementary Figure 1A**). The 5-year EFS for all study patients was 32.9% ± 8.3%. There were 23 events in the entire group; three progressions and nine relapses after GO; and 11 deaths due to infections or treatment-related mortality (**Table 3**; **Supplementary Figure 1B**). The RFS for all patients who achieved CR after GO was 69.6% ± 13%.

**Figure 1:**
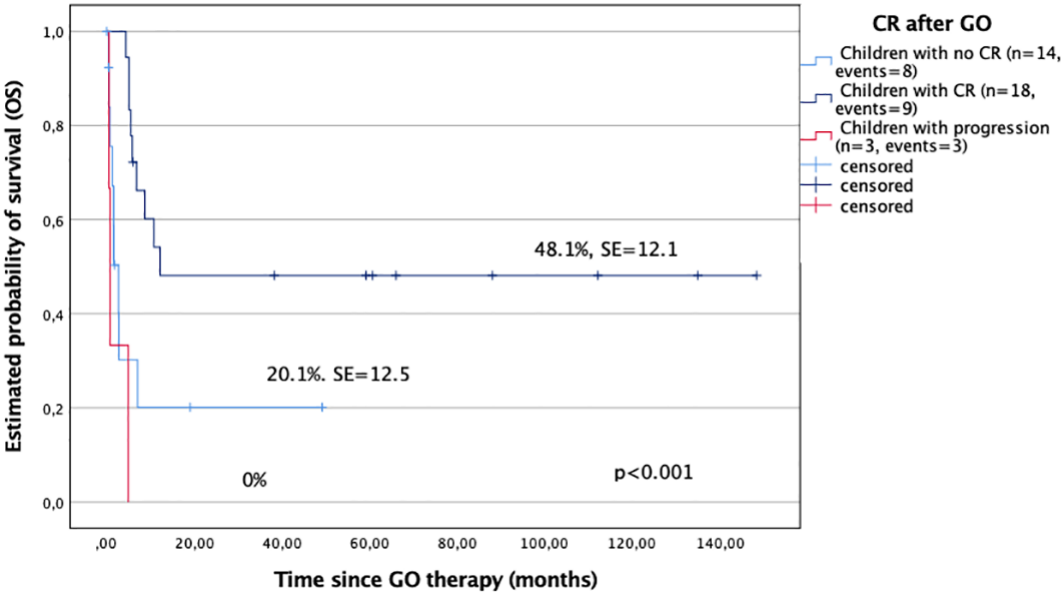
Overall survival (OS) in study children with complete remission after GO (CR) and those who did not respond to GO.

In the analysis, the probability of 5-year OS and EFS after GO treatment for children with CR after GO was significantly better compared with those who did not respond to GO (48.1% ± 12.1% versus 20.1% ± 12.5%; and 48.5% ± 12.1% versus 8.4% ± 8%, respectively), p < 0.001 (**Figure 1**). All patients with progression after GO died.

#### Status of the disease

On the basis of disease status, the best OS probability was observed in children with the first late relapse of 60% ± 21.9%, whereas patients from refractory AML and the second relapse presented poor outcomes [27.3% ± 13.4% and 0% (two of the two deaths), respectively], p = 0.082 (**Supplementary Figure 2**).

#### HSCT after GO

**Figure 2:**
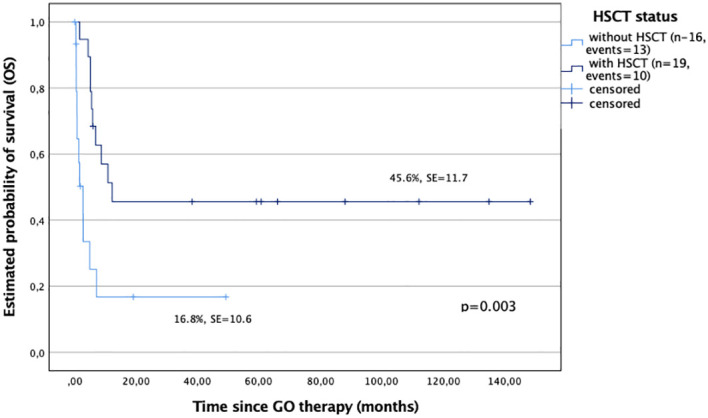
Overall survival (OS) in study children based on the hematopoietic stem cell transplantation (HSCT) procedure followed GO therapy; patients with HSCT after GO therapy (n = 19), and patients without HSCT after GO therapy (n = 16).

All 18 patients who achieved remission after GO had allo-HSCT as a consolidation therapy. A patient without CR after GO had HSCT performed and died soon after due to disease progression. In general, there were 19 transplanted and 16 nontransplanted children in the study cohort (**Table 3**). The OS probability of the children who received allo-HSCT after GO was significantly better compared with those who were not transplanted (45.6% ± 11.7% versus 16.8% ± 10.6%, p < 0.001) (**Figure 2**).

#### Genotype and response to GO

A comparison of the genetic profile based on good versus poor and intermediate risk genetics (no genetic changes or abnormalities with unknown impact on risk (as presented in **Table 1**) showed that children with poor risk genetics had the lowest probability of survival (5-year OS: 16.9% ± 10.9%). Patients with intermediate risk genetics had a better outcome compared with children with poor risk genetics (5-year OS: 41.2% ± 11.9% versus 16.9% ± 10.9%, respectively); however, differences between these groups were not statistically significant (p = 0.377). The best result was observed in children with a good risk profile (n = 4), 50% ± 35.4% (**Supplementary Figure 3**).

#### Age

**Figure 3:**
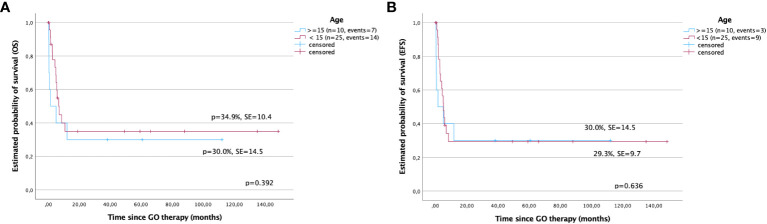
Survival after gemtuzumab ozogamicin (GO) therapy in children based on age. **(A)** Overall survival and **(B)** event-free survival in children under and over 15 years of age.

The 5-year OS was better in children under 15 years compared with those in older children (34.9% ± 10.4% versus 30.0% ± 14.5%, p > 0.05). The EFS was comparable in both groups (29.3% ± 9.7% versus 30.0% ± 14.5%). However, the differences were not statistically significant (p > 0.05) (**Figures 3A, B**).

#### Expression of CD33

**Figure 4:**
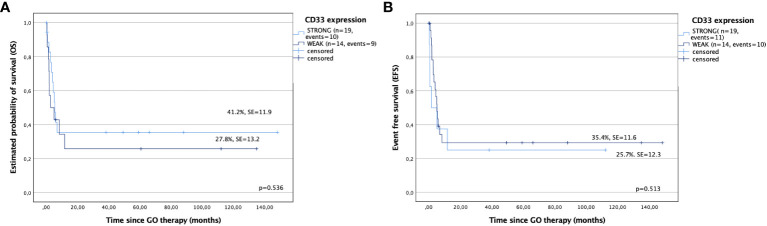
Outcomes after gemtuzumab ozogamicin (GO) therapy based on CD33 expression. **(A)** Overall survival and **(B)** event-free survival in children with strong and weak expression of CD33.

CD33 expression on leukemic cells and its impact on the results were also analyzed. Patients with strong expression of CD33 (more than 50% positive leukemic cells) showed better results compared with patients with weak expression (5-year OS: 41.2% ± 11.9% versus 27.8% ± 13.2%, p = 0.536; 5-year EFS: 35.4% ± 11.6% versus 25.7% ± 12.3%, p = 0.513, respectively); however, the differences were not statistically significant (**Figures 4A, B**). Similarly, relapse rates, estimated by RFS and CIR, were also better for children with strong expression of CD33 compared with that for a cohort with weak CD33 expression (78.8% ± 13.4% versus 60% ± 21.9%, p = 0.799; and 22.2% ± 21.9% versus 40% ± 13.4%, p = 0.799, respectively), however without statistical significance.

## Discussion

The genetic and clinical diversity of AML determines the complexity of the approach to its treatment and continuing need to establish new drugs, including targeted or immune-based therapies. Many studies have been conducted using GO in children and adults to evaluate the efficacy in R/R AML (33–37). GO has been shown to be effective in the setting of R/R AML in adults and children with a rate of CR of up to 30%–35%, whereas, in *de novo*, AML of up to 80–85% (14, 20–23). In the presented study with 35 children with R/R AML in whom GO was administered mainly at a dose of 4.5 mg/m^2^ in multi-agent therapies, in most cases with idarubicin-fludarbine (seven patients) or fludarabine (nine patients), the probability of 5-year OS, EFS, and RFS was 37.1% ± 8.7%, 32.9% ± 8.3%, and 69.6% ± 13%, respectively. The CR rate among all R/R patients reached 51%, and the children who achieved CR after GO had a significantly better outcome compared with those who did not respond; 5-year OS: 48.1% ± 12.1% versus 20.1% ± 12.5%, p < 0.001. As well described in the pediatric literature on R/R AML, also in the presented study, better outcomes were associated with younger age (under 15 years), favorable cytogenetics [CBF and nucleophosmin 1 (NPM1)], late relapse, achievement of the second CR, and the ability to undergo allo-HSCT in CR.

In the report of the BFM study group presented by Niktoreh et al., 76 study children with R/R AML treated with GO at a dose of 2.5–10 mg/m^2^ as a single agent or in combination with other cytostatic agents. Probability of 4-year OS was 18% ± 5% for entire cohort, 27% ± 7% for transplanted, and 0% for non-transplanted children, p < 0.0001 (25). The critical role of allo-HSCT in R/R AML therapy was also confirmed by O’Hear and colleagues; they showed that GO significantly reduced the MRD load before HSCT, without increasing the risk of treatment-related mortality (26). Interestingly, Zahler et al. presented another approach to GO therapy. They evaluated reduced intensity conditioning followed by GO therapy in children with CD33 (≥10% cells positivity). GO was administered more than 60 days after allo-HSCT in two increasing doses. The OS after allo-HSCT and GO in consolidation at 1 and 5 year was 78% and 61%, respectively; and the probability of acute or chronic graft-versus-host disease of grades II to IV were 21% and 33.5%, respectively (29).

The role of GO has also been extensively studied in patients with newly diagnosed AML. As Gamis et al. reported the result of the AAML0531 study with 1,022 enrolled children, adolescents, and young adults, GO significantly improved EFS (3-years EFS: 53.1% versus 46.9%, p = 0.04) with comparable OS. The risk of relapse was significantly reduced among GO recipients, although the remission rate did not improve (88% versus 85%, p = 0.15). More importantly, death in remission was higher in GO recipients (4.2% versus 2.6%, p = 0.09) (24). In our study cohort, the death rate in CR was also high; 11 children died from other causes than treatment failure.

Most of the available meta-analyses of randomized controlled trials with GO refer to adults. It was unequivocally shown that the addition of GO to induction or post-remission chemotherapy significantly improves OS and RFS, decreasing the rate of relapse. Furthermore, the inclusion of GO in conventional chemotherapy cycles improved results mainly in patients with a good-risk genetic profile (37–40). In our study, children with favorable cytogenetics had better OS compared with children with poor genetics (5-year OS: 50% ± 35.4% versus 16.9% ± 10.9%, respectively); however, differences between these groups were not statistically significant (p = 0.377). We confirmed that the genetic profile is one of the important factors; however, because of the low number of study patients, statistical significance was not shown. Children with poor risk genetics, such as FLT3::ITD, complex karyotype, KMT2A::MLLT10, and KMT2A::MLLT3, had worse outcomes compared with good risk genetics or normal or unknown impact genetics. Expectedly, the best result was observed in children with well-established good-risk genetics, such as inv16, t(8;21).

In the literature, an association was reported between high CD33 expression and the NPM1 mutation. The addition of GO to induction in adults with NPM1-mutated AML evaluated in the AMLSG 09-09 trial significantly reduced the CIR compared with patients treated without GO, although an increased early death rate in induction therapy (10.3% and 5.7%, respectively), mainly due to infections (41, 42). Furthermore, children with FLT3::ITD mutations, KMT2A, or single-nucleotide polymorphisms in ABCB1 and CD33 were also more likely to benefit from GO therapy (43, 44). GO added to conventional chemotherapy in patients with poor prognostic molecular markers such as KMT2A and FLT3::ITD improved CR rates and allowed patients to proceed to HSCT (45, 46).

It should be noted that, although GO is a targeted drug, it is generally used in a nontargeted manner. In most studies, patients received GO independently of CD33 expression, and its percentage on leukemic cells was not considered an expected response factor. The correlation between CD33 expression and GO response is ambiguous (27, 32, 47–49). The EORTC-GIMMEMA AML-19 trial showed that GO improved outcomes in patients with AML and CD33 blast expression greater than 80% (47), whereas, in the ALFA-0701 study, a high expression of CD33 in leukemic blasts (more than 30% of cells) did not show any effect on EFS or RFS (32). In our study, there were differences in outcomes between children with AML with strong expression of CD33 (more than 50% positive leukemic cells) and weak expression of CD33 (less than 50%). The first showed better outcomes compared with patients with weak expression (5-year OS: 41.2% ± 11.9% versus 27.8% ± 13.2%, p = 0.536; 5-year EFS: 35.4% ± 11.6% versus 25.7% ± 12.3%, p = 0.513, respectively); however, the differences were not statistically significant due to the low number of patients in the study.

Pollard et al. reported that, in the COG study, children with AML characterized by a high expression of CD33 (75%) had better EFS regardless of the cytogenetic group when given GO but not in a control group (without GO) (48). In the COG AAML0531 trial, different CD33 isoforms detected by splicing a single-nucleotide polymorphism were evaluated with respect to the response to GO therapy. Children with the rs12459419 CC genotype (51% of patients) had a significantly lower risk of relapse and better EFS when treated with GO and chemotherapy (50, 51).

The GO toxicity profile in our study was comparable to toxicities reported in other studies, mainly myelotoxicity and infections. The most common toxicity was grade 3 and 4 neutropenia observed in almost all patients. In earlier studies with GO, a high incidence of SOS, the most serious side effect of GO, was reported. Depending on the study, 6.1%–10% patients developed SOS (37–40, 52, 53). We have not seen a high SOS rate, mainly because most patients received GO in lower doses, 3–4.5 mg/m^2^ compared with those associated with hepatotoxicity (6–9 mg/m^2^). During 14 years of study period, the effectiveness of different doses of GO was reported in the literature, influencing the diversity of doses used in the study; however, after the introduction of the Relapsed AML 2001/01 protocol, GO was usually used at a dose of 4.5 mg/m^2^.

As was shown in adults, GO could also be used in a less intensive manner (28, 29). In our study, GO was administered monotherapy in a heavily pretreated patient with multiple toxicities with the intention of decreasing the disease burden, and, after improving the patient’s condition, we planned to intensify therapy. Unfortunately, the patient died from multiorgan failure. However, GO was successfully used in combination with azacytidine in children with relapsed myeloid leukemia in Down syndrome.

The limitation of the presented study is undoubtedly the small number of patients in all study cohorts that further determined the lack of statistical significance, as well as the nonrandomized and targeted mode of GO administration.

To conclude, as shown, GO in combination with chemotherapy is effective in bridging R/R AML patients to allo-HSCT, with an acceptable toxicity profile, also in children under 15 years of age. The future place of GO in pediatric AML therapies in first-line treatment has yet to be determined in randomized clinical trials designed to identify specific subgroups of pediatric patients who will benefit the most from GO treatment.

## Data availability statement

The original contributions presented in the study are included in the article/**Supplementary Material**. Further inquiries can be directed to the corresponding author.

## Ethics statement

The studies involving humans were approved by The Ethics Committee of Jagiellonian University. The studies were conducted in accordance with the local legislation and institutional requirements. Written informed consent for participation in this study was provided by the participants’ legal guardians/next of kin.

## Author contributions

KP-W: Conceptualization, Data curation, Formal analysis, Investigation, Methodology, Project administration, Supervision, Writing – original draft, Writing – review & editing, Resources, Validation, Visualization. MC: Investigation, Resources, Writing – review & editing, Data curation. SS: Writing – review & editing, Investigation, Resources, Validation. MSu: Investigation, Writing – review & editing. MR: Investigation, Writing – review & editing, Resources. TK: Investigation, Writing – review & editing, Resources. AP: Formal analysis, Writing – review & editing. AW: Investigation, Resources, Writing – review & editing. JS-S: Resources, Writing – review & editing, Investigation. MSa: Resources, Writing – review & editing, Investigation. JW: Resources, Writing – review & editing, Investigation. RC: Resources, Writing – review & editing, Investigation. RT: Investigation, Writing – review & editing, Resources. TS: Investigation, Writing – review & editing, Resources. KZ: Investigation, Writing – review & editing, Resources. TU: Investigation, Writing – review & editing, Resources. MM-H: Investigation, Writing – review & editing, Resources. KK: Investigation, Writing – review & editing, Resources. MK: Investigation, Writing – review & editing, Resources. NI-J: Investigation, Writing – review & editing, Resources. WB: Investigation, Writing – review & editing, Resources, Supervision. KB-S: Conceptualization, Investigation, Writing – review & editing, Methodology, Project administration, Resources.
