# Supplementary material for: Gemtuzumab ozogamicin for relapsed or primary refractory acute myeloid leukemia in children—the Polish Pediatric Leukemia and Lymphoma Study Group experience
Source: Front Immunol. 2023 Dec 22;14:1268993. doi: 10.3389/fimmu.2023.1268993 (PMC10766767; doi:10.3389/fimmu.2023.1268993)

**Supplementary Material**

**Figure 1. Survival after gemtuzumab ozogamicin (GO) treatment**

1. **Overall survival (OS) and (B) event free survival (EFS) in study cohort (n=35 children)**

**A. B.**

**
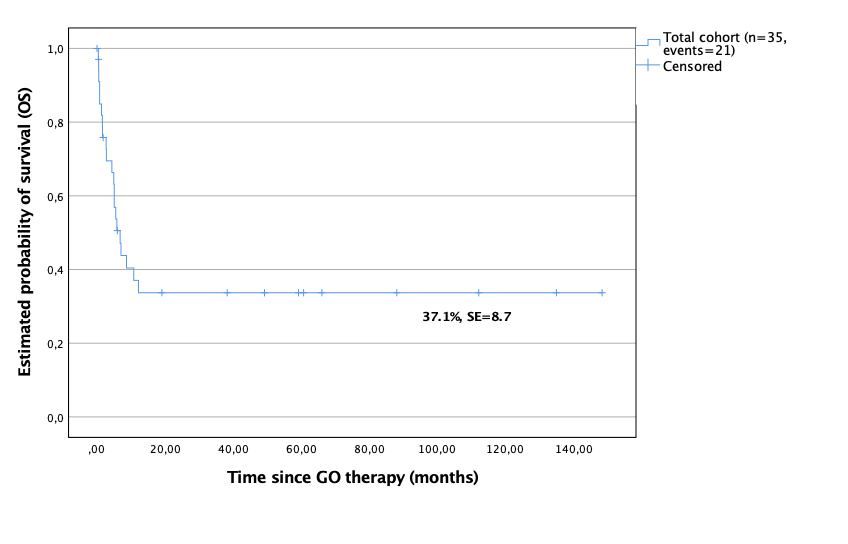

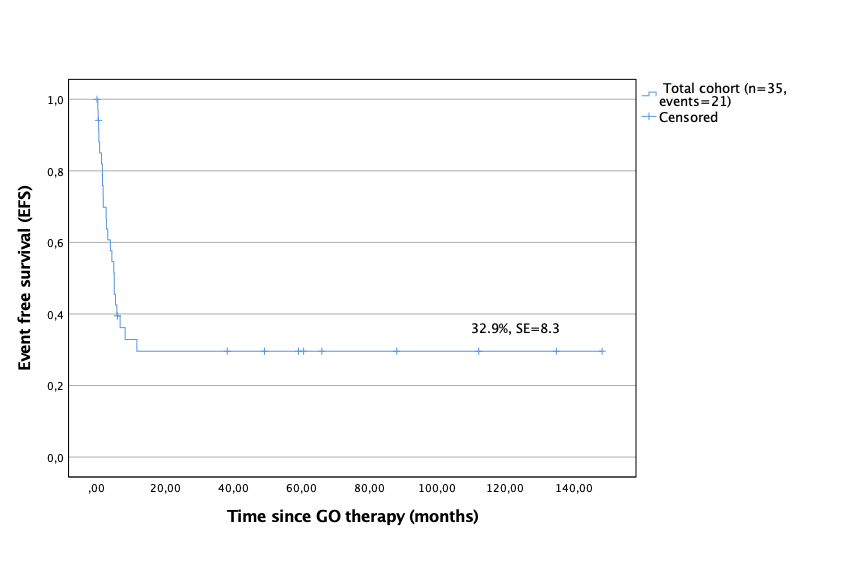
**

**Figure 2. Overall survival (OS) in study children with different disease status; refractory *de novo* AML (n=12), first early relapse (n=16), first late relapse (n=5), second relapse (n=2)**

**
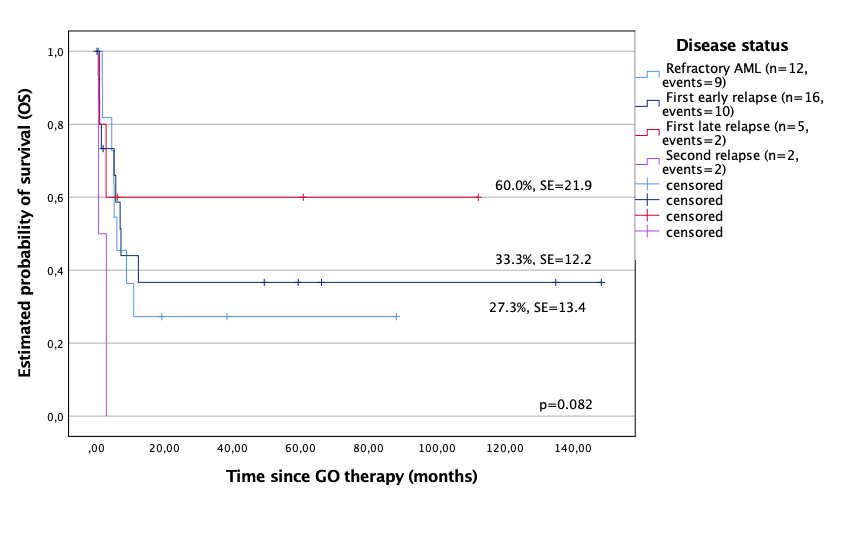
**

**Figure 3. Overall survival after gemtuzumab ozogamicin (GO) based on genetic profile**


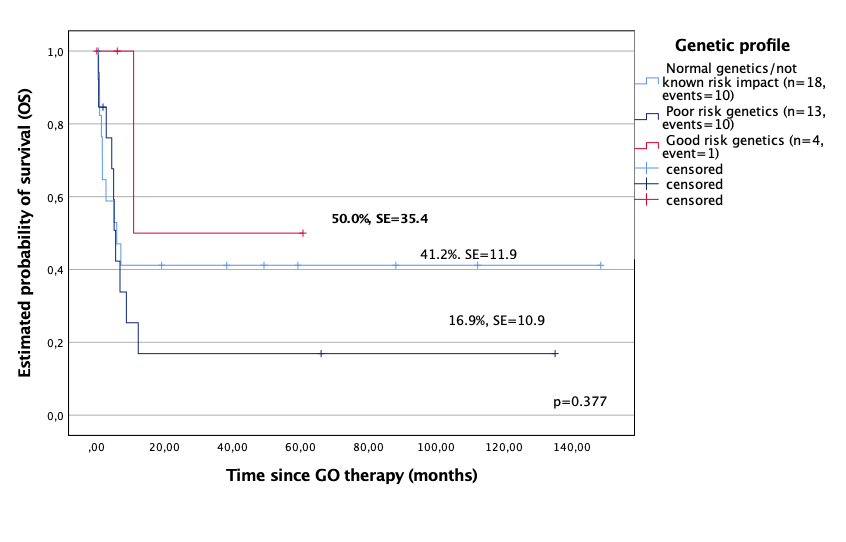

Supplement: Supplementary file 1 [file DataSheet_1.docx]
